# DUODENAL-JEJUNAL BYPASS REDUCES LIPID ACCUMULATION IN THE BROWN ADIPOSE TISSUE OF HYPOTHALAMIC OBESE RATS

**DOI:** 10.1590/0102-672020190001e1497

**Published:** 2020-07-08

**Authors:** Vanessa Marieli CEGLAREK, Zoé Maria GUARESCHI, Gabriela MOREIRA-SOARES, Rafaela Cristiane ECKER-PASSARELLO, Sandra Lucinei BALBO, Maria Lúcia BONFLEUR, Sabrina GRASSIOLLI

**Affiliations:** 1 Endocrine Physiology and Metabolism Laboratory, Center for Biological and Health Sciences, State University of Western Paraná, Cascavel, PR, Brazil; 2 Endocrine Pancreas and Metabolism Laboratory, Department of Structural and Functional Biology, Institute of Biology, Campinas State University, Campinas, SP, Brazil

**Keywords:** Bariatric surgery, Thermogenesis, Obesity, Cirurgia Bariátrica, Termogênese, Obesidade

## Abstract

**Background:**

Thermogenic activity in the brown adipose tissue (BAT) of obese individuals is reduced, and this condition may be modified by bariatric surgery (BS).

**Aim:**

To characterize fat deposition in BAT from hypothalamic obese (HyO) rats submitted to duodenal-jejunal-bypass (DJB) surgery.

**Methods:**

For induction of hypothalamic obesity, newborn male Wistar rats were treated with subcutaneous injections of monosodium glutamate (MSG). The control (CTL) group received saline solution. At 90 days, the HyO rats were submitted to DJB or sham operation, generating the HyO-DJB and HyO-SHAM groups. At 270 days, the rats were euthanized, and the BAT was weighed and submitted to histological analysis.

**Results:**

Compared to BAT from CTL animals, the BAT from HyO-SHAM rats displayed increased weight, hypertrophy with greater lipid accumulation and a reduction in nucleus number. DJB effectively increased nucleus number and normalized lipid deposition in the BAT of HyO-SHAM rats, similar to that observed in CTL animals.

**Conclusion:**

DJB surgery avoided excessive lipid deposition in the BAT of hypothalamic obese rats, suggesting that this procedure could reactivate thermogenesis in BAT, and contribute to increase energy expenditure.

## Graphical Abstract

[Fig f1a]

**Figure f1a:**
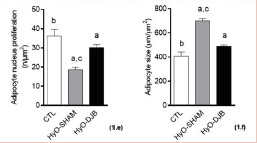
Effects of duodenal-jejunal bypass surgery (DJB) on the proliferation of nuclei and the area of adipocytes in the brown adipose tissue of obese rats

## RESUMO

**Racional:**

A atividade termogênica no tecido adiposo marrom (TAM) de indivíduos obesos encontra-se reduzida, condição que pode ser modificada pela cirurgia bariátrica(CB).

**Objetivo:**

Verificar o efeito da derivação duodeno-jejunal (DDJ) sobre a morfologia do TAM de ratos com obesidade hipotalâmica.

**Métodos:**

Para indução da obesidade hipotalâmica (OHi), ratos Wistar neonatos receberam injeções subcutâneas de glutamato monossódico (MSG). O grupo controle (CTL) recebeu solução salina. Aos 90 dias, os ratos OHi foram submetidos à DDJ (grupo OHi-DDJ) ou a falsa operação (grupo OHi-FO). Aos 270 dias, *eles* foram eutanasiados e o TAM foi pesado e submetido à análise histológica.

**Resultados:**

Em comparação com os animais CTL, o TAM dos ratos OHi-FO apresentou aumento do peso, hipertrofia dos adipócitos com acúmulo de lipídios e redução do número de núcleos. A DDJ reduziu a deposição de gordura e o número de núcleos no TAM de ratos OHi-DDJ em comparação com os OHi-FO, com valores similares aqueles dos animais CTL.

**Conclusões:**

A DDJ foi capaz de evitar a deposição excessiva de lipídios no TAM de ratos com obesidade hipotalâmica, sugerindo que a cirurgia bariátrica poderia reativar a termogênese neste tecido adiposo, contribuindo para aumentar o gasto energético.

## Graphical Abstract

**Figure:**
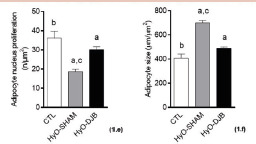
Efeitos da derivação duodenal-jejunal (DDJ) sobre a proliferação de núcleos e área dos adipócitos do tecido adiposo marrom de ratos obesos

## INTRODUCTION

Obesity results from an imbalance between food intake and energy expenditure is regulated by complex physiological mechanisms which involve the brown adipose tissue (BAT)^28^. BAT is primarily characterized by its multilocular adipocytes (elevated number of cytoplasmic lipid droplets), with spherical and slightly eccentric nuclei and huge contents of mitochondria, in which are found high levels of mitochondrial uncoupling protein 1 (UCP-1), responsible for the thermogenic capacity of this tissue^4^. Obese rodents have great fat accumulation in the BAT, with expansion of the adipocyte area and a decrease in mitochondria number, as well as in mitochondrial UCP-1 expression^7^. Therefore, BAT activation may have a protective effect against obesity^12, 26^.

Some studies have shown that the thermogenic activity in BAT can be modulated by bariatric surgery (BS)^2^ ^,^ ^10^ ^,^ ^20^ ^,^ ^21^ ^,^ ^27^. The BS is usually effective for achieving weight loss and energy homeostasis reestablishment in morbidly obese patients^8^ ^,^ ^13^ ^,^ ^18^ ^,^ ^19^ ^,^ ^29^. The duodenal-jejunal bypass (DJB), a procedure that maintains the volume of the stomach, but avoids the passage of food through the duodenum and part of the jejunum, improves glucose and lipids homeostasis^1^ ^,^ ^5^ ^,^ ^11^ ^,^ ^24^, however, the effects of DJB on BAT have never been studied yet.

To study the pathophysiological mechanisms involved in obesity, the neonatal administration of monosodium glutamate (MSG) in rodents is frequently used to induce hypothalamic lesions in these animals, resulting in obesity^3^ ^,^ ^15^. In addition to the excessive fat accumulation and similar to the observed in obese patients, hypothalamic obesity (HyO) rodents^9^ display hyperinsulinemia, insulin resistance and dyslipidemia^5^ ^,^ ^24^.Moreover, these animals present an increase in BAT mass^15^ ^,^ ^17^ and lipid content, and further a reduction in thermogenesis induced by cold^17^. As such, we sought to characterize the effects of DJB on BAT morphology using HyO rats.

## METHOD

### Animals

All experimental procedures were previously approved by the Unioeste’s Animal Ethics Committee (CEUA/November 15/2015). All rats were maintained under controlled luminosity (light 8:00-20:00h) and temperature (22±1° C) and had free access to rodent standard chow (BioBase, SC, Brazil) and water.

### Induction of hypothalamic obesity

Male newborn Wistar rats received one subcutaneous injection per day of monosodium glutamate (4 mg/g body weight) during the first five days of life, (MSG; n=34). During the same period, another group of newborns received an equimolar solution of saline (1.25 mg/g body weight) forming rats control group (CTL, n=17)

### DJB surgery

At 90 days of life, HyO rats were randomly submitted to DJB (HyO-DJB group, n=17) or sham operations (HyO-SHAM, n=17). Preoperative procedures were performed as reported by Meguid et al. (2004)^14^, and the DJB surgery was executed as described by Rubino and Marescaux (2004)^22^. Sham operated rats were submitted to laparotomy and had their intestines massaged without section^24^.

### Histological analysis

At the sixth month after the bariatric procedure, animals’ body weight was registered, rats were euthanized by decapitation. After laparotomy, the BAT was excised and weighed. Subsequently, BAT samples were fixed in 10% formalin for 24 h, dehydrated in alcohol, permeabilized with xylene and then embedded in Paraplast^®^ (Sigma-Aldrich, MO, USA). Sections of 5 µm in thickness were stained with H&E. For the assay, three sections from each BAT were analyzed using a light microscope (Olympus DP71; Tokyo, Japan) with a 40X magnification lens. The Image J software (Bethesda, MD, USA) was used for image analyses. The nuclei proliferation in BAT was verified by counting the number of these ones. For this, a quadrant (501 µm) was selected and the total nuclei in each field was registered. The hypertrophy of adipocytes was evaluated by measuring the adipocytes size (µm). In addition, using the Image J software’s tool “count and measure objects”, the percentage of area occupied by nuclei and fat was evaluated. Additionally, the percentage occupied by the remaining area, which probably represented vascularization, cytosol and extracellular tissue, was calculated and denominated VCE.

### Statistical analysis

Data were analyzed by one-way analysis of variance (ANOVA) followed by the Tukey post-test (p<0.05), using GraphPad Prism software (GraphPad Inc., CA, USA).

## RESULTS

**FIGURE 1:**
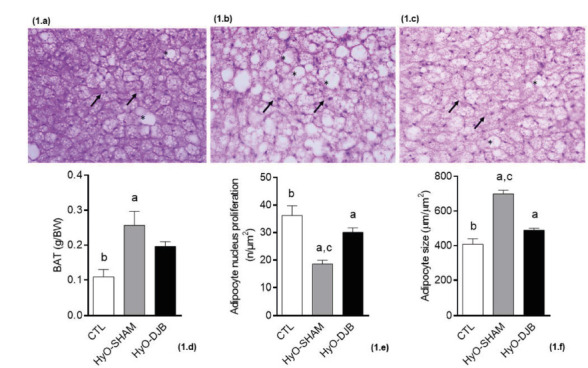
Representative photomicrography of BAT (H&E 40x): a) CTL; b) HyO-SHAM; c) HyO-DJB; d) BAT weight; e) adipocyte nucleus proliferation; f) adipocyte size.

The body weight of the HyO-SHAM and HyO-DJB rats were approximately 25% lower than that of the CTL group (p<0.0001, Table 1). In addition, no difference in body weight was observed between the HyO-DJB and HyO-SHAM rats. Figure 1 shows the effects of DJB on BAT in HyO rats and its histological aspects. The BAT weight was 135.1% greater in HyO-SHAM animals, compared to CTL rats (p<0.0084, Figure 1d). DJB surgery did not affect the weight of this fat depot (Figure 1d) in relation to both other groups, CTL and HyO-SHAM.

Black arrows indicate adipocyte nuclei; “*” Indicates lipiddroplets; different letters above the bars represent statistical differences between the groups. One-way ANOVA with Tukey post-test (p< 0.05, n=5-6)

**TABLE 1:** Body weights of CTL, HyO-SHAM and HyO-DJB rats

Group	Mean ± SEM (g)
CTL	470.30 ± 9.45 ^a^
HyO-SHAM	355.80 ± 13.83 ^b^
HyO-DJB	347.10 ± 8.40 ^b^

Data are means±SEM (n=6-8 rats); different letters represent statistical differences between the groups. One-way ANOVA with Tukey post-test (p< 0.05)

Histological analyses showed that in the BAT of the CTL group presented characteristics of multilocular adipose tissue, since adipocyte cells contained small lipid droplets of different sizes. In contrast, in HyO-SHAM animals, the cells in the BAT were expanded and displayed a higher fat content, almost ceasing to be multilocular and becoming unilocular Differently, in BAT of HyO-DJB group contained some fat droplets, but the cells were more similar to those of the CTL group. The spherical nucleus of cells is located centrally or eccentrically in all groups, despite being reduced in the HyO-SHAM BAT. In addition, the cytoplasm of the BAT cells of the CTL group appeared to contain numerous mitochondria and a rich supply of capillaries between the cells, since these regions were stained with hematoxylin (in purple). However, in the BAT of HyO-SHAM animals these regions have been reduced, while in HyO-DJB BAT it was similar to that of CTL rats.

HyO-SHAM BAT presented a reduction of 48.74% in nucleus number (Figure 1e; p<0.0001) and a larger (71.11%) adipocyte size, in relation to the BAT of CTL rats (Figure 1f; p<0.0001). Interestingly, in BAT from HyO-DJB animals, an increase of 62.16% in nucleus number was observed, when compared to BAT from the HyO-SHAM group (Figure 1e; p<0.0001), no significant differences from that observed in the CTL group. Adipocyte size in the BAT of HyO-DJB rats was similar to that observed in the BAT of CTL rats (Figure 1f).

**FIGURE 2:**
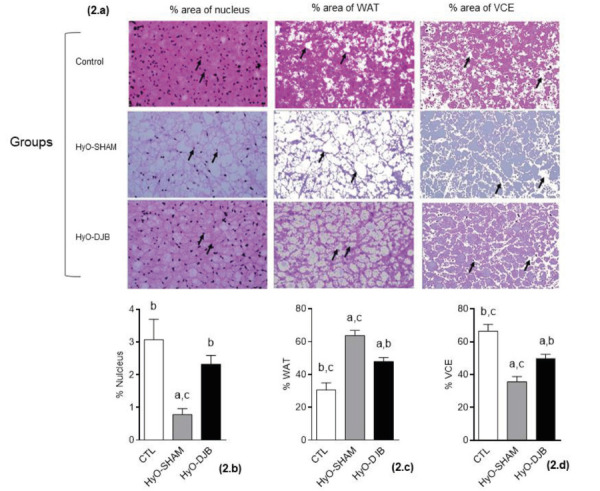
Representative photomicrography of BAT (H&E 40x 50.0µm scale): the columns represent % of nucleus occupation; % of WAT occupation; % of VCE, respectively. Black arrows in the first column indicate adipocyte nuclei; in the second indicate adipocyte in WAT; in the third indicate BAT.

Figure 2a demonstrates the effects of DJB surgery on the percentages of nuclei, WAT and VCE occupation per field in the BAT of CTL and HyO rats. The percentage of area occupied by nuclei in HyO-SHAM BAT was approximately 76% lower in relation to the same parameter in BAT from CTL rats (Figure 2b; p<0.0012). The percentage area of nuclei in the HyO-DJB BAT was nearly 197% higher than in the BAT of the HyO-SHAM group (p<0.0012, Figure 2b), statistically resembling the CTL group. The percentage of fat content per field in the BAT from the HyO-SHAM group was 109% and 32% higher than the fat percentage content found in the BAT from CTL and HyO-DJB groups, respectively (Figure 2c, p<0.0001). However, the area occupied by lipids in HyO-DJB BAT remained 57% greater, when compared to the fat percentage area in BAT from CTL animals (Figure 2c; p<0.0001). Consequently, the percentage of VCE area in the BAT from HyO-SHAM rats was 46.49% and 28.73% lower, respectively, in relation to the same area in BAT from CTL and HyO-DJB rats (Figure 2d; p<0.0001). HyO-DJB BAT also presented reduced percentage (25%) in the VCE area, compared to the BAT from CTL rats (p<0.0001, Figure 2d).

Graphs=means±SEM; different letters above the bars represent statistical differences between the groups. One-way ANOVA with Tukey post-test (p< 0.05, n=5-6)

## DISCUSSION

Obesity due to lesions in the hypothalamus has negative impacts on survival and quality of life of patients and BS can represent a therapeutic alternative for this syndrome^9^. Herein, using MSG obese rats to mimic hypothalamic obesity syndrome, we demonstrated that adipocytes of BAT from HyO rats lost the multilocular droplets lipids profile and presented a reduction in nucleus number and augment in fat content. For the first time, we observed that at six months after DJB operation, the BAT morphology in HyO-DJB rats returned to a similar morphology to that of BAT from CTL animals.

BAT is an important site of cold-induced non-shivering thermogenesis^28^. The sympathetic nervous system (SNS) is responsible for activating lipolysis and fatty acid ß-oxidation in BAT. Therefore, the proton gradient generated by this process is diverted to ATP-synthase through UCP-1, and the energy generated is dissipated as heat^4^. Reductions in SNS activity and UCP1 expression contribute to lower energy expenditure and higher adiposity in BAT^7^. HyO mice exhibited hypertrophy of BAT with an 85% increase in wet weight and lipid content and did not mobilize BAT lipids after cold exposure to 4º C for 6 h^17^. Another study showed a reduction in GLUT^4^ transporter levels in BAT from HyO rats^15^. Additionally, the type II thyroxine 5-deiodinase (T2) activity in BAT from HyO mice was reduced after cold and norepinephrine stimulation^25^. A decrease in retroperitoneal sympathetic nerve activity and lower adrenal catecholamine stores have also been reported in HyO mice^23^. As such, modifications in BAT morphology in the obese rats, observed in the present study, may be due to the low SNS activity associated with norepinephrine stimulation reduction, which could alter the function of BAT in HyO animals.

Currently, BS is frequently used as a treatment in morbidly obese patients^13^.^.^ However, there are few studies showing the effects of BS on BAT and these reports present contrasting data regarding the surgery’s benefits^2^ ^,^ ^10^ ^,^ ^20^ ^,^ ^21^ ^,^ ^27^. Obese subjects showed increased non-shivering thermogenesis in BAT one year after surgery-induced weight loss, demonstrating that BAT can be recruited after bariatric procedures in humans^27^. Additionally, BS displayed a beneficial impact on the metabolic activity of BAT in morbidly obese patients^2^. The increase in brown/beige adipose tissue activity related to surgery-induced weight loss occurs independently of changes in hypothalamic activity^20^ and BAT activity was found to be increased in obese non-diabetic and unchanged in obese diabetic subjects submitted to bariatric operation^21^. 

Mice submitted to BS by several techniques presented increased BAT thermogenesis, mediated by higher levels of growth hormone and insulin growth factor 1^6^. On the other hand, no significant difference was observed in BAT volume at 6 and 12 months after bariatric procedures in patients with morbid obesity^10^.

No study has demonstrated the effects of bariatric procedure in the BAT from HyO model. This obesity disorder is caused by damage to the hypothalamus, leading to metabolic and endocrine disturbances. Traditional treatments of obesity are not effective for patients with this disturbance^9^. Using MSG-treated rats as an experimental model to study HyO, our group has demonstrated that DJB surgery ameliorates glucose homeostasis and insulin sensitivity, normalizes pancreatic islet function and decreases islet-cell proliferation, as well as improving lipid profile and hepatic steatosis^1^ ^,^ ^5^ ^,^ ^24^. In the present study, we contribute a little more to the understanding of the effects of DJB surgery on Hy obesity. We, herein, observed lower lipid accumulation in BAT, an increase in nucleus number and the reestablishment of the percentage of area occupied by nuclei in HyO-DJB. Taken together, these results suggest that DJB surgery in HyO animals had a proliferative effect on BAT and could elevate the thermogenic activity in this adipose tissue, probably by normalizing insulin levels and sensitivity, through ameliorating the SNS tonus. 

## CONCLUSION

DJB procedure in HyO rats reduces lipid accumulation and adipocyte size and increases nucleus number in BAT, suggesting reactivation of BAT thermogenesis. The morphological changes induced by DJB surgery in the BAT of obese rats reflects the enhancement of BAT metabolic capacity.
